# Supplementary material for: Inferring the number of COVID-19 cases from recently reported deaths
Source: Wellcome Open Res. 2020 Apr 27;5:78. [Version 1] doi: 10.12688/wellcomeopenres.15786.1 (PMC7255910; doi:10.12688/wellcomeopenres.15786.1)
Supplement: Peer review history

# Peer review: Reviewer response for version 1

**DOI:** 10.21956/wellcomeopenres.17312.r38540

**Published:** 2020-05-26

**Authors:** Camille Pelat

**Affiliations:** 1 Santé publique France, Saint-Maurice, France

This article describes a statistical modeling method for estimating the number of COVID-19 cases from the first reported deaths in a defined location. The described methodology can provide useful information for decision making, especially as a Shiny app has been developed for facilitating quick application of the method by public health practitioners, and the R code has been made available.

**Introduction:**

- I would be interested to see in the text a few words about how many (and which) countries found themselves in the situation of observing no COVID-19 case before the first deaths were reported. The reference provided (number 2) is not really specific about this point.

 

**Methods:**

- The statistical method is well described and seems sound.
- I have a minor comment: in practice, published estimates of the CFR and R will be used as input parameters for the model. These estimates are derived from samples and are usually published with a certain measure of uncertainty, typically the standard deviation or a confidence interval. My understanding is that this estimation uncertainty on these input parameters is not taken into account in the prediction model: instead, the CFR and R are held constant for all simulations drawn with a set of parameter.
- Taking into account the uncertainty on these input parameters may lead to even greater prediction intervals, but may reflect more completely the uncertainty about the total number of cases given the current knowledge about the disease at a certain point in time. This could be done, for example, by drawing the CFR in a Beta distribution with a and b derived from the published mean and sd instead of holding it constant. In the Shiny app, the user could provide the confidence interval.

 

**Discussion:**

- It would be interesting if the authors could comment on the availability of other published methods developed for inferring the number of cases based on reported deaths. If such methods exist, how do they compare in their approach and results with the proposed one? What are the comparative strengths of the proposed method?
- typo error: “theunderlying size…”

Is the rationale for developing the new method (or application) clearly explained?

Yes

Is the description of the method technically sound?

Yes

Are the conclusions about the method and its performance adequately supported by the findings presented in the article?

Yes

If any results are presented, are all the source data underlying the results available to ensure full reproducibility?

Yes

Are sufficient details provided to allow replication of the method development and its use by others?

Yes

Reviewer Expertise:

biostatistics, public health

I confirm that I have read this submission and believe that I have an appropriate level of expertise to confirm that it is of an acceptable scientific standard.

# Peer review: Reviewer response for version 1

**DOI:** 10.21956/wellcomeopenres.17312.r38539

**Published:** 2020-05-19

**Authors:** Benjamin M. Bolker

**Affiliations:** 1 Department of Mathematics and Statistics, McMaster University, Hamilton, ON, Canada

This is a useful, technically correct, and clearly written contribution.

 Could the authors comment on how much extra mileage one gets/advantages of this approach relative to simply saying that the current number of cases is approximately equal to 1/CFR? That is, does one have to reconstruct the past history to know how much trouble one is currently in?

 What would the effect of a heterogeneous CFR be? (I believe this would correspond e.g. to a 'beta-Geometric distribution', unless one instead wanted to treat it as a finite mixture of probabilities for discrete risk categories).

 It would be nice to have a little more detail (i.e. a few sentences) on the simulation procedure. I see how to get from CFR and deaths to a total number of preceding cases, and how to simulate times of symptom onset for the observed deaths. It's not completely obvious to me how to get from there to 'history of past cases' (i.e. incidence over time); does one run the renewal process backward in time? Or use branching-process theory to find the time distribution of symptom onset of the index case given the current size of the epidemic?

 Please clarify "We assume that past cases caused secondary transmissions independently (i.e. are not ancestral to each other), so that simulated cases for each death can be added." Does this mean that you assume that all observed deaths are from separate lineages/transmission chains? (The last sentence of the paragraph suggests that, but the initial statement could probably be clearer.) (Does this assumption even matter if we are in the branching-process regime?).

 I appreciate that the authors are trying to keep things simple, and thus the scenario-based approach (try the model for a range of CFR/R values and see what is implied) is useful. I note that the confidence intervals are already very wide (that's part of the point), but there are several quantities that are treated as known (delay distribution, serial interval distribution); I wonder how sensitive the results are to these assumptions (probably not much - I'm guessing that with R specified they might only change the timing, not the numbers). Given that the authors are already basing the answers on 25,000 solutions, it might not be too hard to construct point estimates and intervals based on a prior/uncertainty distribution of R and CFR (rather than constructing separate scenarios), and allowing for uncertainty in the delay and serial distributions.

Minor comments/typos:

- Intro, line 1; methods, l. 7: extra comma inside parens before superscript refs?)
- "use [a] different CFR for each case"
- "parameters" values
- "theunderlying"
- "schoolclosures"
- In tables 1 and 2 consider stating "2.5% quantile, 25% quantile, 50% quantile, 97.5% quantile" (rather than lower/upper x 95%/50%) ?

Is the rationale for developing the new method (or application) clearly explained?

Yes

Is the description of the method technically sound?

Yes

Are the conclusions about the method and its performance adequately supported by the findings presented in the article?

Yes

If any results are presented, are all the source data underlying the results available to ensure full reproducibility?

Yes

Are sufficient details provided to allow replication of the method development and its use by others?

Yes

Reviewer Expertise:

ecology, evolution, epidemiological modeling

I confirm that I have read this submission and believe that I have an appropriate level of expertise to confirm that it is of an acceptable scientific standard.
